# Biology of Aging I Antisense Oligonucleotide-Mediated SRF Reduction Ameliorates Mitochondrial Dysfunction in Cardiac Aging

**DOI:** 10.1093/geroni/igaf122.2536

**Published:** 2025-12-31

**Authors:** Pankaj Patyal, Gohar Azhar, Xiaomin Zhang, Ambika Verma, Jeanne Wei

**Affiliations:** University of Arkansas for Medical Sciences, Little Rock, Arkansas, United States; University of Arkansas for Medical Sciences, Little Rock, AR, LITTLE ROCK, Arkansas, United States; University of Arkansas for Medical Sciences, Little Rock, Arkansas, United States; University of Arkansas for Medical Sciences, Little Rock, Arkansas, United States; University of Arkansas for Medical Sciences, Little Rock, AR, LITTLE ROCK, Arkansas, United States

## Abstract

Cardiac aging is characterized by progressive mitochondrial dysfunction, contributing to heart failure and other age-related cardiovascular diseases. Serum response factor (SRF) is a key transcription factor regulating cardiac integrity, but its elevated expression in aging myocardium worsens mitochondrial dysfunction and heart pathology. This study examines whether antisense oligonucleotide-mediated SRF reduction can alleviate mitochondrial dysfunction in cardiac aging. A cardiac-specific antisense SRF transgenic mouse model with modestly reduced SRF levels was generated. Mice aged 12–15 months were analyzed for cardiac changes, including histology, mitochondrial function, and oxidative stress. Histological analysis revealed reduced cardiac hypertrophy and fibrosis in Anti-SRF Tg mice. Ultrastructural analysis showed well-preserved mitochondrial structure, increased mitochondrial density (p < 0.01), and more cristae (p < 0.01). Mitochondrial DNA copy number was also higher (p < 0.05). Western blot analysis showed increased expression of mitochondrial regulators PGC-1α (p < 0.01), NT-PGC-1α (p < 0.05), PGC-1β (p < 0.05), and OPA1 (p < 0.05), with reduced Drp1 (p < 0.05). High-resolution respiratory functional analysis by Oroboros O2K revealed increased activity of mitochondrial complexes I-IV (p < 0.01 to p < 0.001). This is further corroborated by western blot analyses of OXPHOS (I–V) which shows that SRF reduction resulted in upregulation of mitochondrial complex proteins of electron transport chain. SRF reduction also reduced oxidative stress, indicated by lower 4-HNE levels (p < 0.01) and increased MnSOD expression. In conclusion, cardiac-specific SRF reduction mitigates hypertrophy, fibrosis, mitochondrial dysfunction, and oxidative stress, suggesting its potential as a therapeutic target for cardiac aging.

